# Processing of Self versus Non-Self in Alzheimer’s Disease

**DOI:** 10.3389/fnhum.2016.00097

**Published:** 2016-03-08

**Authors:** Rebecca L. Bond, Laura E. Downey, Philip S. J. Weston, Catherine F. Slattery, Camilla N. Clark, Kirsty Macpherson, Catherine J. Mummery, Jason D. Warren

**Affiliations:** Dementia Research Centre, UCL Institute of Neurology, University College LondonLondon, UK

**Keywords:** Alzheimer’s, self processing, voice, source memory, Interpersonal Reactivity Index, tactile

## Abstract

Despite considerable evidence for abnormalities of self-awareness in Alzheimer’s disease (AD), the cognitive mechanisms of altered self-processing in AD have not been fully defined. Here we addressed this issue in a detailed analysis of self/non-self-processing in three patients with AD. We designed a novel neuropsychological battery comprising tests of tactile body schema coding, attribution of tactile events to self versus external agents, and memory for self- versus non-self-generated vocal information, administered in conjunction with a daily life measure of self/non-self-processing (the Interpersonal Reactivity Index). Three male AD patients (aged 54–68 years; one with a pathogenic mutation in the Presenilin 1 gene, one with a pathogenic mutation in the Amyloid Precursor Protein gene, and one with a CSF protein profile supporting underlying AD pathology) were studied in relation to a group of eight healthy older male individuals (aged 58–74 years). Compared to healthy controls, all patients had relatively intact tactile body schema processing. In contrast, all patients showed impaired memory for words previously presented using the patient’s own voice whereas memory for words presented in other voices was less consistently affected. Two patients showed increased levels of emotional contagion and reduced perspective taking on the Interpersonal Reactivity Index. Our findings suggest that AD may be associated with deficient self/non-self differentiation over time despite a relatively intact body image: this profile of altered self-processing contrasts with the deficit of tactile body schema previously described in frontotemporal dementia associated with *C9orf72* mutations. We present these findings as a preliminary rationale to direct future systematic study in larger patient cohorts.

## Introduction

The processing of self in relation to cognitive and bodily states and the external environment (in particular, other people) has emerged as an important theme in neurodegenerative disease. ‘Self’ is a multi-component construct which (in neuropsychological terms) entails a number of cognitive operations. From first principles, these operations are likely to include the maintenance of perceptual and conceptual boundaries between the self and non-self (the world at large, including other people), internalization of an appropriate self-image, and updating of that image based on experience. Shaping of the self-image and regulation of one’s own emotional and social behavior depend on understanding of one’s own mental states, how others relate to self and the impact of self on others: these processes in turn involve awareness and interpretation of others’ feelings and beliefs in relation to one’s own (‘theory of mind’: Mehta et al., 2014; Sommer et al., 2014; Bradford et al., 2015; Cheng et al., 2015; Simm et al., 2015; van der Weiden et al., 2015), while emotional contagion is a basic mechanism for synchronizing one’s own physiological and behavioral states to those of others, captured using measures such as the Inter-personal Reactivity Index (IRI; assessing the extent to which an individual experiences self- versus non-self oriented feelings in emotionally charged social situations) (Davis, 1980; Sturm et al., 2013). Despite much recent interest, the effects of neurodegenerative diseases on the component operations of self-processing have not been explored in detail. Loss of self-awareness is a leading feature of the frontotemporal dementias, an important and heterogeneous group of non-Alzheimer dementias frequently characterized by abnormal social and emotional behaviors and underpinned by selective disintegration of distributed neural circuitry in the frontal and anterior temporal lobes (Warren et al., 2013). Impaired self-processing in these diseases manifests as anosognosia and deficits of self-knowledge, self-monitoring and self-conscious emotions such as embarrassment (Eslinger et al., 2005; Rankin et al., 2005; Sturm et al., 2006; Banks and Weintraub, 2008). In addition, deficits of body schema representation and self/non-self differentiation have been linked to somatic delusions and other neuropsychiatric disturbances in patients with frontotemporal dementia caused by pathogenic expansions in the *C9orf72* gene (Downey et al., 2012, 2014). It is increasingly recognized that impaired self-processing is not restricted to frontotemporal dementia but also plays a central role in other neurodegenerative diseases, notably Alzheimer’s disease (AD), the most common dementia of later life. Patients with AD may exhibit reduced awareness of their own behavior, personality, mood and cognitive deficits (Kashiwa et al., 2005; Rankin et al., 2005; Banks and Weintraub, 2008; Zamboni et al., 2013), and a heightened propensity to react to the emotions of those around them (emotional contagion) (Sturm et al., 2013), raising the possibility that self/non-self boundaries may become more permeable in AD. Reduced understanding of one’s own mental states (impaired theory of own mind) has been linked to an attenuated sense of self in AD (Simm et al., 2015). More fundamentally, AD has been associated with impaired body schema processing and loss of self-recognition (Grewal, 1994; Mozaz and Morris, 1997; Connors and Coltheart, 2011), these features becoming more prominent as the disease evolves. However, the cognitive mechanisms that underpin abnormalities of self-processing in AD have not been fully defined.

Aside from their clinical relevance, deficits of self-processing may inform our understanding of the systems pathophysiology and molecular mechanisms of neurodegenerative diseases. In the frontotemporal dementias, anosognosia and lack of self-knowledge are underpinned by disintegration of predominantly anterior fronto-insular ‘salience’ and temporal ‘appraisal’ networks (Sollberger et al., 2014) while body schema deficits in association with *C9orf72* mutations may be more specifically attributable to dysfunction of a cortico-thalamo-cerebellar network (Mahoney et al., 2012; Downey et al., 2014). Deficits of self-processing in AD may reflect the involvement of areas associated with the so-called ‘default mode network’ (Buckner and Carroll, 2007; Sturm et al., 2013; Zamboni et al., 2013; Cosentino et al., 2015). This network comprises distributed mesial temporal, parietal and prefrontal areas and is likely to be integral to the pathogenesis of AD: the network is targeted early by the pathological process, and involvement of the network accounts for the impairment of episodic memory that is typically a leading feature of AD (Warren et al., 2012). However, the functions of this network continue to be defined: named for its association with stimulus-independent thought in the awake resting brain, the network has also been implicated in various active neural operations besides autobiographical memory in the healthy brain and in AD, including tracking of auditory information streams, homeostasis, sensorimotor imagery, understanding others’ mental states (theory of mind) and empathy (Goll et al., 2012; Beissner et al., 2013; Li et al., 2014; Ge et al., 2015; Golden et al., 2015; Hyatt et al., 2015). These functions are unlikely to engage the network uniformly and may depend on the interaction of particular ‘default mode’ components with connected elements of other brain networks. Collectively, this diverse functional profile would position the default mode network at the interface of the internal milieu with the external sensory and social world (Dixon et al., 2014; Metzak et al., 2015). It is therefore plausible that this network plays a central role in the regulation of self-directed versus non-self-directed cognition, and that AD should disrupt this role. While the separable brain network substrates of frontotemporal dementia, AD, and other dementias reflect the distinct molecular pathologies of these diseases, imperfect correlation between pathology and phenotype remains a major nosological and clinical issue (Warren et al., 2012, 2013). Accordingly, there is considerable interest in identifying novel behavioral signatures of altered self-processing in dementias with defined pathology: such signatures might illuminate molecularly specified network mechanisms that generate and maintain the interface between self and non-self and at the same time, suggest novel clinical biomarkers for detecting and tracking these diseases.

Here we addressed this issue in detailed case studies of three patients with a typical clinical syndrome of AD, associated with either a pathogenic mutation or a suggestive profile of cerebrospinal fluid (CSF) neurodegeneration markers. Although patients with genetically mediated (autosomal dominant) AD tend to be younger than those with the more common sporadic form of the disease, studying these individuals offers substantial advantages: besides molecular definition *per se*, such patients potentially illustrate AD-associated processes without the confounding effects of cerebrovascular and other comorbidities that occur more commonly in the elderly. In this study, patients were assessed in relation to healthy older individuals using a neuropsychological battery that probed tactile body (self) schema coding and attribution of tactile events to self versus external agents using previously described procedures (Downey et al., 2012, 2014) and a novel test comparing self- and non-self-generated memories for vocal information. Participants’ neuropsychological performance was assessed in conjunction with a questionnaire that probes processing of self in relation to others in daily life and has been shown previously to capture alterations of inter-personal reactivity in AD (Sturm et al., 2013). Based on previous clinical and functional neuroanatomical evidence, we hypothesized that (relative to healthy older controls) AD is associated with reduced ability to differentiate self from non-self-generated events, particularly where these must be updated from memory; and that this in turn leads to heightened inter-personal reactivity on daily life metrics. We present our findings in a small number of AD cases, as a preliminary exploration that we hope will motivate further work.

## Materials and Methods

### Participants and General Assessments

Three male patients (aged 54–68 years) who fulfilled current consensus clinical criteria for AD (Dubois et al., 2007) with genetic or CSF corroboration were recruited via our tertiary cognitive disorders clinic and eight healthy older males (aged 58–74 years) with no history of neurological or psychiatric illness were identified from a local research database. Participant characteristics are summarized in **Table 1.** All participants were of British origin and spoke with a standard southern English accent; none had a history of clinically relevant hearing loss. All participants underwent a comprehensive general neuropsychological assessment, which corroborated the clinical classification (**Table 1**). Patients all had a clinical diagnosis of mild to moderately severe AD led by decline in episodic memory with supervening symptoms of executive and/or posterior cortical dysfunction; and in addition, on volumetric T1-weighted brain MRI exhibited typical neuroanatomical features of AD, namely disproportionate bilateral hippocampal and mesial temporal lobe atrophy with minimal associated vascular burden. Two patients had familial (autosomal dominant) AD on the basis of confirmed pathogenic mutations, Case 1 in the *APP* (Amyloid Precursor Protein) and Case 2 in the *PSEN1* (Presenilin 1) gene (details in **Table 1**). The remaining patient (Case 3) screened negative for pathogenic mutations but had a CSF profile of neurodegeneration marker proteins supporting underlying AD pathology (total tau 466 pg/L, beta-amyloid_1-42_ 298 pg/L; ratio 1.6, normal < 0.8) based on local laboratory reference ranges.

**Table 1:** General demographic, clinical and neuropsychological data for participants.

Characteristic	Healthy control group	Alzheimer’s disease		
		
		Case 1	Case 2	Case 3
Genetic mutation		*APP* V717l	*PSEN1* R269H	–
n/Gender	8 Males	Male	Male	Male
Age (years) (range)	66.4 (6.2) (58 – 74)	**54 (*z* = -1.99)**	68 (*z* = 0.26)	65 (*z* = -0.22)
Education (years)	16.3 (1.5)	14 (*z* = -1.51)	16 (*z* = -0.17)	15 (*z* = -0.84)
Symptom duration (years)	N/A	11	6	9
MMSE (/30)	N/A	20	22	20
**General intellect**				
WASI Verbal IQ	127 (6.2)	116 (*z* = -1.75)	**107 (*z* = -3.20)**	**91 (*z* = -5.77)**
WASI Performance IQ	128 (8.1)	***105 (z = -2.84)***	***89 (z = -4.82)***	***81 (z = -5.81)***
NART (/50)	42.1 (4.7)	39 (*z* = **-**0.66)	40 (*z* = -0.45)	42 (*z* = -0.03)
**Episodic memory**				
RMT Faces^∗^	1.07 (0.87)	0.45 (*z* = -0.70)	**-2.98 (*z* = -4.63)**	**-0.95 (*z* = -2.30)**
RMT Words^∗^	1.05 (0.56)	**-*2.41 (z = -6.20)***	**-*2.62 (z = -6.58)***	**-*5.39 (z = -11.55)***
**Executive skills**				
WASI Matrices (/32)	27.4 (2.3)	24 (*z* = 1.47)	**14 (*z* = 4.15)**	**11 (*z* = 4.78)**
WASI Block Design (/71)	49.0 (9.3)	38 (*z* = -1.18)	**23 (*z* = -2.79)**	**25 (*z* = -2.58)**
WMS-R digit span forward (/12)	9.6 (2.3)	9 (*z* = -0.27)	7 (*z* = -1.13)	8 (*z* = -0.70)
WMS-R digit span reverse (/12)	8.8 (1.6)	6 (*z* = -1.74)	6 (*z* = -1.74)	6 (*z* = -1.74)
Stroop ink color (sec)	52 (12.1)	**83 (*z* = 2.58)**	**114 (*z* = 5.14)**	64 (*z* = 1.02)
**Verbal skills**				
WASI Vocab (/80)	73 (4.4)	66 (*z* = **-**1.57)	**57 (*z* = -3.62)**	**56 (*z* = -3.84)**
WASI Similarities (/48)	40 (1.6)	40 (*z* = **-**0.08)	**35 (*z* = -3.12)**	**26 (*z* = -8.60)**
GNT (/30)	26 (2.8)	**20 (*z* = 1.96)**	**19 (*z* = 2.20)**	26 (*z* = 0.20)
BPVS (/150)	148 (1.9)	147 (*z* = -0.52)	148 (*z* = 0.00)	**143 (*z* = -2.59)**
**Posterior cortical skills**				
GDA (/24)	16.5 (5.9)	12 (*z* = -0.77)	N/A	12 (*z* = -0.77)
VOSP Object Decision (/20)	19.0 (0.8)	19 (z = 0.00)	18 (*z* = -1.32)	**15 (*z* = -5.29)**


*Group mean (standard deviation) performance scores are reported for the healthy control group; for individual patients, raw scores are presented along with *z*-scores (in parentheses) indexing that patient’s performance on neuropsychological tests relative to the control group (individual *z*-score > 1.96 standard deviations beyond healthy control mean in bold) and where individual cases consistently showed a deficit, significant group differences (*p* < 0.05) are indicated with italics (see text). Maximum scores for neuropsychological tests where appropriate are in parentheses. ^∗^*z*-scores based on published norms. *APP*, amyloid precursor protein gene; BPVS, British Picture Vocabulary Scale (Dunn et al., 1982); GDA, Graded Difficulty Arithmetic (Jackson and Warrington, 1986); GNT, Graded Naming Test (McKenna and Warrington, 1983); MMSE, Mini Mental State Examination (Folstein et al., 1975); N/A, not available; NART, National Adult Reading Test (Nelson, 1982); *PSEN1*, Presenilin 1 gene; RMT, Recognition Memory Test (Warrington, 1984); RMT, Recognition Memory subtests of the Camden Memory Tests (Warrington, 1996); VOSP, Visual Object and Spatial Perception Battery (Warrington and James, 1991); WASI, Wechsler Abbreviated Scale of Intelligence (Wechsler, 1999); WMS-R, Wechsler Memory Scale-Revised (Wechsler, 1987).*

This study was approved by the local institutional ethics committee, and all participants gave written informed consent in accordance with the Declaration of Helsinki.

### Rating of Inter-Personal Reactivity

We administered three subscales of the IRI (Davis, 1980): Perspective Taking (the individual’s tendency to consider situations from others’ points of view), Empathic Concern (how much the individual tends to care about how other people feel), and Personal Distress (how the individual reacts to intensely emotional social situations); the remaining subscale (Fantasy) was not completed, as it contains items dependent on tracking narratives and therefore is potentially vulnerable to impaired episodic memory in AD. Each subscale contains seven items (each rated 0 [does not describe him/her well] to 4 [describes him/her very well], maximum score 28 per subscale; see Supplementary Material). Healthy control participants completed the questionnaire first-hand, while patients’ scores were obtained from their caregivers using a modified third-person version of the questionnaire, a procedure shown previously to index patient personality characteristics reliably (Strauss et al., 1993). Our rationale in obtaining caregiver reports was to avoid the potentially confounding limitation of impaired recollection in self-person accounts from patients with AD.

### Assessment of Tactile Self-Schema Processing

#### Tactile Two-Point Discrimination Task

The experimental procedure used for this test was adapted from a previously described procedure (Lenzenweger, 2000). Manual tactile two-point discrimination thresholds were determined using a standard clinical two-point aesthesiometer lightly applied along the transverse axis of each participant’s dominant palm. Prior to commencing the test, it was established that each participant could easily detect the touch of the aesthesiometer. During the test, the participant was seated comfortably and blindfolded, and the task on each trial was to indicate whether one or two points (applied simultaneously) had been detected. Both ascending and descending series were administered. In a descending series, the distance between the two points was incrementally reduced in 5 mm steps from an initial separation of 35 mm, then at 3, 2, and 1 mm, until the participant indicated that ‘one point’ was detected on two consecutive trials; the first of these successive ‘one point’ responses was taken as the two point detection threshold for that descending series. In an ascending series, the distance between the two points was incrementally increased in 5 mm steps after initial trials at 1, 2, 3, and 5 mm, until the participant indicated that two points were detected on two consecutive trials; the first of the successive ‘two points’ responses was taken as the two-point detection threshold for that ascending series. Descending and ascending series were each repeated three times, yielding a total of six threshold estimates for each participant; a mean two-point discrimination threshold was calculated by averaging the threshold scores across all six series, and these individual participant mean two-point thresholds were incorporated in subsequent analyses of group tactile threshold differences.

#### Rubber Hand Illusion

Body ownership was assessed using a procedure adapted from the rubber hand illusion paradigm of Botvinick and Cohen (1998). The participant was seated comfortably at a table wearing latex gloves; an identical glove filled with water (the ‘rubber hand’) was placed visibly on the table alongside the participant’s dominant hand, which was itself obscured by a partition. Both the participant’s hidden dominant hand and the rubber hand were stroked synchronously using paintbrushes for 3 min while the participant watched the rubber hand. The participant then completed a questionnaire (see Supplementary Material) to assess the presence and extent of any somatosensory illusion during stimulation: responses were graded using a 7-point Likert scale (1 signifying a strong percept; 7 signifying no percept; highest possible score across target items: 21).

#### Self/Non-Self Tactile Attribution

Attribution of a tactile stimulus to one’s self versus another person was assessed using a previously described procedure (Downey et al., 2012, 2014; schematic of experimental set-up in **Supplementary Figure S1**). A paintbrush was suspended using a cross-clamp from a rod positioned between two table-mounted retort stands, such that it could be rotated freely by manipulating a handle at one end. The blindfolded participant was positioned with the dominant hand resting palm down on the table between the retort stands, and the apparatus was adjusted so that the paintbrush lightly tracked across the skin of the hand when rotated. On each trial, the handle was rotated by the participant using the non-dominant hand, and the paintbrush was randomly moved along the suspended rod between trials, so that the brush would either contact the participant’s hand (‘self’ condition) or would not contact the participant’s hand (‘non-self’ trials); on ‘non-self’ trials, the experimenter delivered the tactile stimulus by using an identical paintbrush, either in time with the participant’s own handle action (synchronous condition) or with a short delay (around 1 s; asynchronous condition). The task on each trial was to decide whether the tickle stimulus was generated by the participant’s own action or by that of the experimenter. Thirty randomly ordered trials were administered (10 self, 10 non-self synchronous, 10 non-self asynchronous).

### Assessment of Self/Non-Self Voice Memory

A novel computerized paradigm was adapted from the Recognition Memory Test for Words (RMTW) (Warrington, 1984) to probe whether recognition memory for spoken words or source memory for speaker varied according to the agent responsible for the auditory verbal percept between participant groups. This task was programmed using Matlab 2013a^®^ and is schematised in **Supplementary Figure S2**. Word stimuli were the first 48 RMTW items, randomly assigned to one of three conditions each comprising 16 trials: ‘self’ (the participant’s own voice), ‘non-self: same gender’ (other male) and ‘non-self: opposite gender’ (female). Non-self stimuli were pre-recorded by male and female speakers using a standard southern English accent; variation of gender was designed to manipulate the speaker’s vocal similarity to the participant across conditions. During the study (encoding) phase of the task, participants wore headphones while written words were presented sequentially on a computer monitor controlled by the experimenter; the task on each trial was to read the word aloud and while reading the participant heard either their own voice or another speaker’s voice through the headphones (Audio-Technica ATH-M50), presented at a comfortable listening level (at least 70 dB, effectively masking any percept from bone conduction of own voice); participants’ own voices were delivered via auditory feedback (using PsychPortAudio within Psychtoolbox for Matlab, and a Yeti USB microphone) with an imperceptible (∼16 ms) delay, while other voices were delivered with a fixed delay of 800 ms after presentation of the written word, determined empirically to coincide with mean latency to voice onset when reading aloud in normal older individuals and patients with AD (Gold et al., 2005). In the subsequent test (retrieval) phase immediately following the encoding phase, recognition memory for previously presented words was assessed with the two-alternative forced choice procedure on the written words (one target and one foil per trial) used in the standard RMTW. In addition, source memory for speaker was assessed by asking the participant on each trial to identify which voice had previously spoken the target word (self, other male, other female). All participants were first familiarized with the procedure using practice trials not subsequently administered in the test proper, and it was established that each participant could easily discriminate between the three voices presented.

Participant responses were recorded for offline analysis. Recognition memory performance was analyzed as the proportion of trials on which the participant selected the correct word, while voice source memory performance was recorded as the proportion of successful recognition trials on which the participant also attributed words to the correct agent.

### Statistical Analysis

Data were analyzed using SPSS v22^®^. In order to demonstrate the degree to which patients’ data on variables of interest deviated from the distribution of values associated with the healthy control group, we calculated individual patient *z*-scores (where *z* = (individual score-control mean)/control standard deviation); these *z*-scores were based on transformed data for variables where raw control data did not approximate a normal distribution (see **Tables 1** and **2**). Due to the small sample size, non-parametric tests were also run on raw data to substantiate any apparently consistent group differences or where floor or ceiling effects precluded use of *z*-scores (Mann–Whitney U; see Tables); one-sample Wilcoxon signed-rank tests were used to compare group performance means to chance-level performance in each voice memory task condition. A statistical threshold of *p* < 0.05 was taken as the criterion of significance for all tests; for individual patients, this would correspond to *z*-score > 1.96 standard deviations beyond the healthy control mean.

**Table 2:** Experimental task performance data for participants.

Experimental measure	Healthy control group	Alzheimer’s disease		
		
		Case 1	Case 2	Case 3
**Inter-personal reactivity index**				
Perspective Taking (/28)	17.1 (3.7)	16 (*z* = -0.31)	**2 (*z* = -4.11)**	**6 (*z* = -3.02)**
Empathic Concern (/28)	19.3 (4.4)	22 (*z* = 0.63)	15 (*z* = -0.97)	14 (*z* = -1.19)
Personal Distress (/28)	9.6 (6.1)	14 (*z* = 0.72)	**23 (*z* = 2.20)**	**23 (*z* = 2.20)**
**Tactile discrimination**				
Threshold (mm)	11.0 (2.3)	7.5 (*z* = -1.51)	15.0 (*z* = 1.73)	14.2 (*z* = 1.37)
**Rubber hand illusion**				
Mean score target items (/7)^∗^	5.8 (0.9)	**2.7 (*z* = -3.41)**	7.0 (*z* = 1.38)	5.7 (*z* = -0.09)
**Self/non-self touch attribution**				
Self (/10)	9.8 (0.7)	10	10	9
Non-self synchronous (/10)	4.5 (2.6)	2	10	4
Non-self asynchronous (/10)	9.1 (1.2)	9	10	9
**Self/non-self voice memory †**				
Overall recognition	0.83 (0.10)	0.67 (*z* = -1.56)	**0.63 (*z* = -1.96)**	**0.44 (*z* = -3.77)**
Own voice	0.84 (0.09)	**0.56 (*z* = -3.18)**	**0.56 (*z* = -3.18)**	**0.56 (*z* = -3.18)**
Non-self: same gender	0.82 (0.12)	0.88 (*z* = 0.46)	0.75 (*z* = -0.60)	**0.38 (*z* = -3.78)**
Non-self: opposite gender	0.82 (0.15)	0.56 (*z* = -1.65)	0.56 (*z* = -1.65)	**0.38 (*z* = -2.66)**
Overall attribution	0.36 (0.05)	0.38	0.20	0.38
Own voice	0.48 (0.11)	0.22	0.56	0.33
Non-self: same gender	0.31 (0.08)	0.43	0.08	0.50
Non-self: opposite gender	0.29 (0.18)	0.44	0.00	0.33


*Group mean (standard deviation) performance scores are reported for the healthy control group; for individual patients, raw scores are presented along with *z*-scores (in parentheses) indexing each patient’s performance on neuropsychological tests relative to the control group (individual *z*-score > 1.96 standard deviations beyond healthy control mean in bold). *z*-scores are not displayed for the source attribution tasks (action and voice memory) as floor or ceiling effects rendered their use inappropriate. See text for further details. Maximum scores for neuropsychological tests where appropriate are in parentheses. ^∗^Likert scale 1 = no percept to 7 = strong percept. ^b^bbData in the self/non-self voice memory task are presented as proportion of items in each condition correct (chance level performance 0.5 for recognition task, 0.33 for attribution task).*

## Results

Performance profiles of participants (AD cases and healthy controls) on experimental tests are summarized in **Table 2.**

### Interpersonal Reactivity Index

Case 1 did not deviate significantly from the range of healthy control scores on any of the three IRI subscales (|*z*|≤ 0.72). By contrast, relative to healthy controls Cases 2 and 3 showed a reduced tendency to consider situations from others’ points of view (Perspective Taking: *z* = -4.11, -3.02 respectively), and an increased tendency to become distressed when observing others in adverse circumstances (Personal Distress: *z* = 2.20, 2.20) despite similar levels of empathic concern toward others (*z* = -0.97, -1.19).

### Tactile Self-schema Processing

On the tactile two-point discrimination task, none of the AD cases showed a threshold significantly different from controls (all |*z*|≤ 1.73). On the rubber hand task, Case 1 reported a significantly stronger illusory percept than healthy controls (*z* = -3.41) while Cases 2 and 3 did not (*z* = 1.38, -0.09). In the tactile attribution task, a ceiling effect on ‘self’ trials precluded transformation of these performance data to a normal distribution appropriate for the use of *z*-scores. However, in a non-parametric group-wise analysis, compared to the healthy control group the AD cases collectively showed no significant deficit on any dimension of tactile self-schema processing (all tests non-significant with Mann–Whitney *U* ≥ 9.5, *p* ≥ 0.545).

### Self/Non-Self Voice Memory

For the adapted RMTW task, relative to healthy controls all three AD cases showed a deficit for recognizing words in the self condition (words heard in own voice; all *z* = -3.18), In the non-self male (same gender) voice condition, Cases 1 and 2 performed similarly to healthy controls (*z* = 0.46, -0.60) while Case 3 showed a deficit (*z* = -3.78). In the non-self female (opposite gender) voice condition, relative to healthy controls Cases 1 and 2 again showed no significant deficit (*z* = -1.65) while Case 3 showed a deficit (*z* = -2.66); it should be noted that wide control group variance in this condition may have limited our power to detect significant individual case deficits here.

For the voice source attribution task, a *post hoc* (Wilcoxon signed-rank) analysis revealed that healthy controls performed significantly above chance for attribution of words previously heard in their own voice but not in either non-self condition (self condition, *p* = 0.012; non-self conditions, *p* ≥ 0.482). AD cases considered collectively did not perform significantly above chance in any condition (*p* ≥ 0.593 for all conditions). The overall relatively poor performance of healthy controls on this task rendered the use of individual *z*-scores unreliable; however, in a non-parametric group-wise analysis, AD cases collectively showed no significant deficit of voice attribution accuracy across conditions, relative to the healthy control group (Mann–Whitney *U* ≥ 5.5, *p* ≥ 0.183).

## Discussion

Here we have shown that patients with a diagnosis of autosomal dominant or sporadic AD may have altered cognitive and behavioral responses in processing self-associated versus non-self-associated information. Relative to healthy older individuals, all three patients with AD studied here had reduced recognition memory for self-generated vocal information whereas recognition of non-self-generated vocal information was less consistently affected. There was the further suggestion that patients with AD (unlike healthy controls) may be unable to self-attribute their own previous vocal events. All patients showed relatively intact representation of self-schema and retained differentiation of self- from non-self-generated actions based on tactile cues; this contrasts with the impairment of body schema processing previously documented in patients with *C9orf72* mutations (Downey et al., 2012, 2014). In addition, all patients here were reported to show levels of empathic concern in daily life comparable to healthy controls, but individual patients showed a reduced tendency to assume another’s perspective and increased levels of distress in emotionally intense situations: these findings corroborate previous evidence for heightened emotional contagion in clinically diagnosed AD (Sturm et al., 2013) but in the present context also suggest impaired ability to shift between self- and non-self-oriented behaviors.

A plausible interpretation of our findings is that patients with AD may have difficulty in retaining self- versus non-self-generated events over time (and therefore, difficulty integrating those events into a dynamic cognitive representation of the self) despite a relatively intact body image. Such a deficit would be consistent with previously documented impairments of cognitive self-monitoring, self-awareness and source memory in AD (Rosa et al., 2014; Rosen et al., 2014; Lehrner et al., 2015; Perrotin et al., 2015) and might contribute to the failure of patients with AD to benefit from self-referenced cueing and enactment when performing memory tasks (Lalanne et al., 2013; Rosa et al., 2014). Deficient differentiation of previously experienced self- versus non-self-generated events might also provide a candidate mechanism for both heightened emotional contagion and reduced perspective shifting exhibited by AD patients in everyday social situations. Vocal stimuli may be a sensitive vehicle with which to probe self/non-self boundaries in AD, given the propensity of the culprit default mode network to generate both inner speech and verbal hallucinations (Alderson-Day et al., 2015) and the role of this network in tracking auditory events (Goll et al., 2012; Golden et al., 2015). Though episodic memory for spoken words has been less widely studied than memory for words presented visually, normal subjects have been shown to recognize previously presented auditory verbal material and speakers accurately and episodic memory for auditory verbal items is influenced by speaker characteristics (Geiselman and Glenny, 1977; Palmeri et al., 1993; Goh, 2005). The generally poor voice source memory of the present healthy control group may reflect their older age range, since it has also been shown that memory for both auditory verbal and vocal features declines substantially with age (Pilotti and Beyer, 2002). This poor overall control performance is likely to have obscured any condition effect in AD due to varying similarity of the other speaker’s characteristics (here, their gender) to the patient’s own voice.

The cognitive mechanisms that mediate theory of mind and the regulation of emotional inter-personal behavior, though not explicitly addressed in this study, are relevant to any consideration of impaired self-processing in AD. On cognitive and neurobiological grounds, these processes are likely to interact: interpreting others’ mental states and understanding the effects of one’s own behavior on others facilitate the modulation of self-image and self boundaries through experience and self-other integration (Mehta et al., 2014; Sommer et al., 2014; Bradford et al., 2015; Cheng et al., 2015; Simm et al., 2015; van der Weiden et al., 2015). Moreover, theory of mind and emotion regulation are targeted by neurodegenerative diseases and derangements thereof contribute to clinical symptoms in AD and other dementias (Goodkind et al., 2010; Bora et al., 2015; Simm et al., 2015). Although theory of mind impairment in AD has generally been deemphasised in relation to frontotemporal dementia (Bora et al., 2015), it is possible that patients with AD may have more specific deficits in interpreting their own mental states linked to defective self-processing (Simm et al., 2015). The present study suggests that this may in part reflect impaired access to one’s own prior mental states and behavior, a mechanism previously proposed to contribute to impaired emotion regulation in AD (Goodkind et al., 2010). Impaired emotion regulation may in turn have contributed to the altered inter-personal reactivity observed in our AD patients.

Though neuroanatomical correlation was not possible in this study, the performance profile of our patient group suggests candidate neural substrates for impaired self-processing in AD. The finding of impaired episodic memory for self-generated material is consistent with involvement of the default mode network previously implicated both in the pathogenesis of AD and in autobiographical memory and other self-referential cognitive operations (Buckner and Carroll, 2007; Goll et al., 2012; Beissner et al., 2013; Li et al., 2014; Ge et al., 2015; Golden et al., 2015; Hyatt et al., 2015). The sparing of tactile body schema coding in the present patients further suggests that the brain network substrate for altered self-processing in AD may be differentiated from the cortico-thalamo-cerebellar network substrate previously proposed to underpin the body schema deficit exhibited by patients with frontotemporal dementia due to *C9orf72* mutations (Mahoney et al., 2012; Downey et al., 2014). Separable signatures of abnormal self-processing in AD and frontotemporal dementia could reflect both the relative extent of damage within particular networks and altered interactions between networks at key hub zones such as the insula (Zhou and Seeley, 2014; Cosentino et al., 2015). Viewed from another perspective, self-processing may be a particularly relevant paradigm for assessing network function in diseases such as AD, precisely because dynamic, distributed network mechanisms are required to support it. Functional neuroimaging (in particular, connectivity based) techniques are likely to be required to elucidate these mechanisms. However, the present data do not resolve the specificity of voice memory deficits for AD versus other neurodegenerative pathologies, since other diseases have yet to be assessed using this paradigm.

The small number of cases is a clear limitation of this study and we therefore regard the present data as preliminary, providing a rationale and a practical methodology to direct future work. The present data indicate wide individual variation between patients on a number of measures of self-processing: any conclusions regarding an AD-associated signature must therefore remain heavily qualified. However, our findings suggest a program for a systematic analysis of self-processing in AD. First, there is a need to study larger patient cohorts, ideally including both sporadic cases (with corroborating CSF or amyloid-ligand brain imaging) representing the phenotypic spectrum of AD and genetically determined cases of AD, ultimately with histopathological correlation. Larger case numbers (recruited collaboratively across specialist centers) would enable a more fine-grained analysis of different pathogenic mutations causing AD with potentially separable neuroimaging and behavioral signatures (Scahill et al., 2013). Patients with AD should be compared directly with frontotemporal dementia and other neurodegenerative diseases using a hierarchy of cognitive tests sampling elementary sensory coding, integrative, evaluative and mnestic dimensions of self-processing. The interface between self-processing, theory of mind and the regulation of emotional inter-personal behavior should be defined; candidate instruments have been identified (Simm et al., 2015). In addition, first-hand accounts from patients will be required to assess explicit awareness of own behavior in AD: ideally these should be collected in parallel with caregiver reports, which are likely to yield complementary information. Only by studying a range of component behavioral processes and by comparing diseases directly will it be possible to determine the syndromic and molecular specificity of identified abnormalities of self-processing and its component sub-processes. The underlying brain mechanisms of cognitive disease signatures will only be captured by correlative neuroanatomical methods that can detect shifts in neural network function and connectivity. The rational application of functional neuroimaging techniques will in turn require paradigms that have been designed to address specific hypotheses about altered self-processing in particular diseases, informed by both behavioral evidence and theoretical models of self-processing (a candidate neural architecture that could support template-based self/non-self differentiation has recently been proposed: Clark and Warren, 2015). A further priority for future work will be to study disease cohorts longitudinally. Longitudinal analysis would allow deficits to be assessed at different disease stages, as it is likely that abnormalities of self-processing evolve over time; for example, the present data might be reconciled with previous evidence for abnormal body schema coding in AD if body schema abnormalities emerge relatively later in the course of this disease (Grewal, 1994; Mozaz and Morris, 1997; Connors and Coltheart, 2011). In addition, only by studying patients longitudinally will it be possible to assess the sensitivity of self-processing abnormalities as putative disease biomarkers: autosomal dominant AD, though accounting for only a very small fraction of total disease burden is of disproportionate importance in this enterprise, since mutation carriers present the unique opportunity to assess complex functions such as self-processing at the very earliest disease stages and before onset of clinical symptoms. We hope the present observations will inform such a future program of work to assess the impact of AD and other neurodegenerative diseases on the processing of self in relation to others and the implications of altered self-awareness for patients’ daily lives.

## Author Contributions

RB, LD, and JW made substantial contributions to the conception and design of the work. RB, LD, PW, CS, KM, CC, and CM were involved in data acquisition and analysis. All authors were involved in drafting or revising the paper critically for important intellectual content, gave approval for the final version to be published and agree to be accountable in ensuring that questions related to the accuracy or integrity of any part of the work are appropriately investigated and resolved.

## Conflict of Interest Statement

The authors declare that the research was conducted in the absence of any commercial or financial relationships that could be construed as a potential conflict of interest.
