# Supplementary material for: Processing of Self versus Non-Self in Alzheimer’s Disease
Source: Front Hum Neurosci. 2016 Mar 8;10:97. doi: 10.3389/fnhum.2016.00097 (PMC4781858; doi:10.3389/fnhum.2016.00097)

SUPPLEMENTARY MATERIAL

Self/non-self processing in Alzheimer's disease, by RL Bond et al

Interpersonal Reactivity Index questionnaire (adapted from Davis, 1980)

Question	Score	Subscale
I often have tender, concerned feelings for people less fortunate than me		EC
I sometimes find it difficult to see things from the "other guy's" point of view		PT
Sometimes I don't feel sorry for other people when they are having problems		EC
In emergency situations, I feel apprehensive and ill-at-ease		PD
I try to look at everybody's side of a disagreement before I make a decision		PT
When I see someone being taken advantage of, I feel kind of protective toward them		EC
I sometimes feel helpless when I am in the middle of a very emotional situation		PD
I sometimes try to understand my friends better by imagining how things look from their perspective		PT
When I see someone get hurt, I tend to remain calm		PD
Other people's misfortunes do not usually disturb me a great deal		EC
If I'm sure I'm right about something, I don't waste much time listening to other people's arguments		PT
Being in a tense emotional situation scares me		PD
When I see someone being treated unfairly, I sometimes don't feel very much pity for them		EC
I am usually pretty effective in dealing with emergencies		PD
I am often quite touched by things that I see happen		EC
I believe that there are two sides to every question and try to look at them both		PT
I would describe myself as a pretty soft-hearted person		EC
I tend to lose control during emergencies		PD
When I'm upset at someone, I usually try to "put myself in his shoes" for a while		PT
When I see someone who badly needs help in an emergency, I go to pieces		PD
Before criticizing somebody, I try to imagine how I would feel if I were in their place		PT

The participant was asked to rate each question by assigning a score to each statement between 0 (does not describe me well) and 4 (describes me very well); instructions were adapted for completion by patients' caregivers. For reference, the subscale for each question is also indicated here: PT, Perspective Taking, EC (Empathic Concern), Personal Distress (PD) (see text for details).

Questionnaire to assess rubber hand illusion (adapted from Botvinick & Cohen, 1998)

<u>It seemed as if I were feeling the touch of the paintbrush in the location where I saw the rubber hand touched.</u>

1	2	3	4	5	6	7
**Strongly Agree**						**Strongly Disagree**

<u>It seemed as though the touch I felt was caused by the paintbrush touching the rubber hand.</u>

1	2	3	4	5	6	7
**Strongly Agree**						**Strongly Disagree**

<u>I felt as if the rubber hand were my hand.</u>

1	2	3	4	5	6	7
**Strongly Agree**						**Strongly Disagree**

<u>It felt as if my (real) hand were drifting towards the rubber hand.</u>

1	2	3	4	5	6	7
**Strongly Agree**						**Strongly Disagree**

<u>It seemed as if I might have more than one hand or arm on the side of my body where the rubber hand was.</u>

1	2	3	4	5	6	7
**Strongly Agree**						**Strongly Disagree**

<u>It seemed as if the touch I was feeling came from somewhere between my own hand and the rubber hand.</u>

1	2	3	4	5	6	7
**Strongly Agree**						**Strongly Disagree**

<u>It felt as if my (real) hand were turning ‘rubbery’.</u>

1	2	3	4	5	6	7
**Strongly Agree**						**Strongly Disagree**

<u>It appeared (visually) as if the rubber hand were drifting towards my (real) hand.</u>

1	2	3	4	5	6	7
**Strongly Agree**						**Strongly Disagree**

<u>The rubber hand began to resemble my own (real) hand, in terms of physical appearance.</u>

1	2	3	4	5	6	7
**Strongly Agree**						**Strongly Disagree**

The first three items of the questionnaire are the ‘target’ items used in analysis; others are catch’ items, included to probe for any particularly suggestible participants. None of the participants described here appeared to be overly suggestible, always scoring a mean of more than 5 across the catch items.

Figure S1: Schematic of the self/non-self touch attribution paradigm (adapted from Downey et al., 2014)

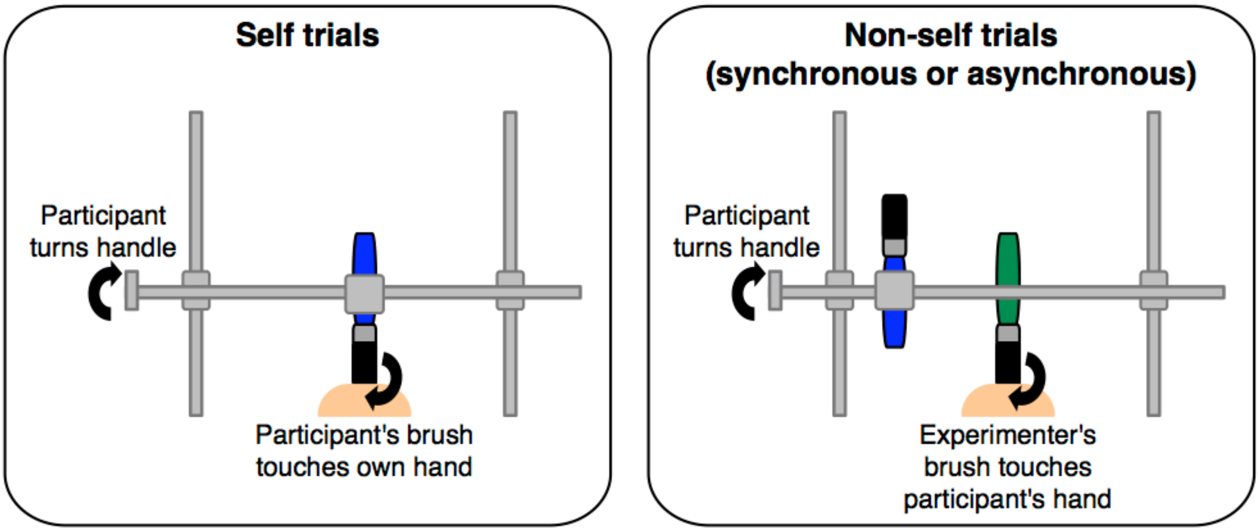

Figure S2: Schematic of the self/non-self voice memory paradigm

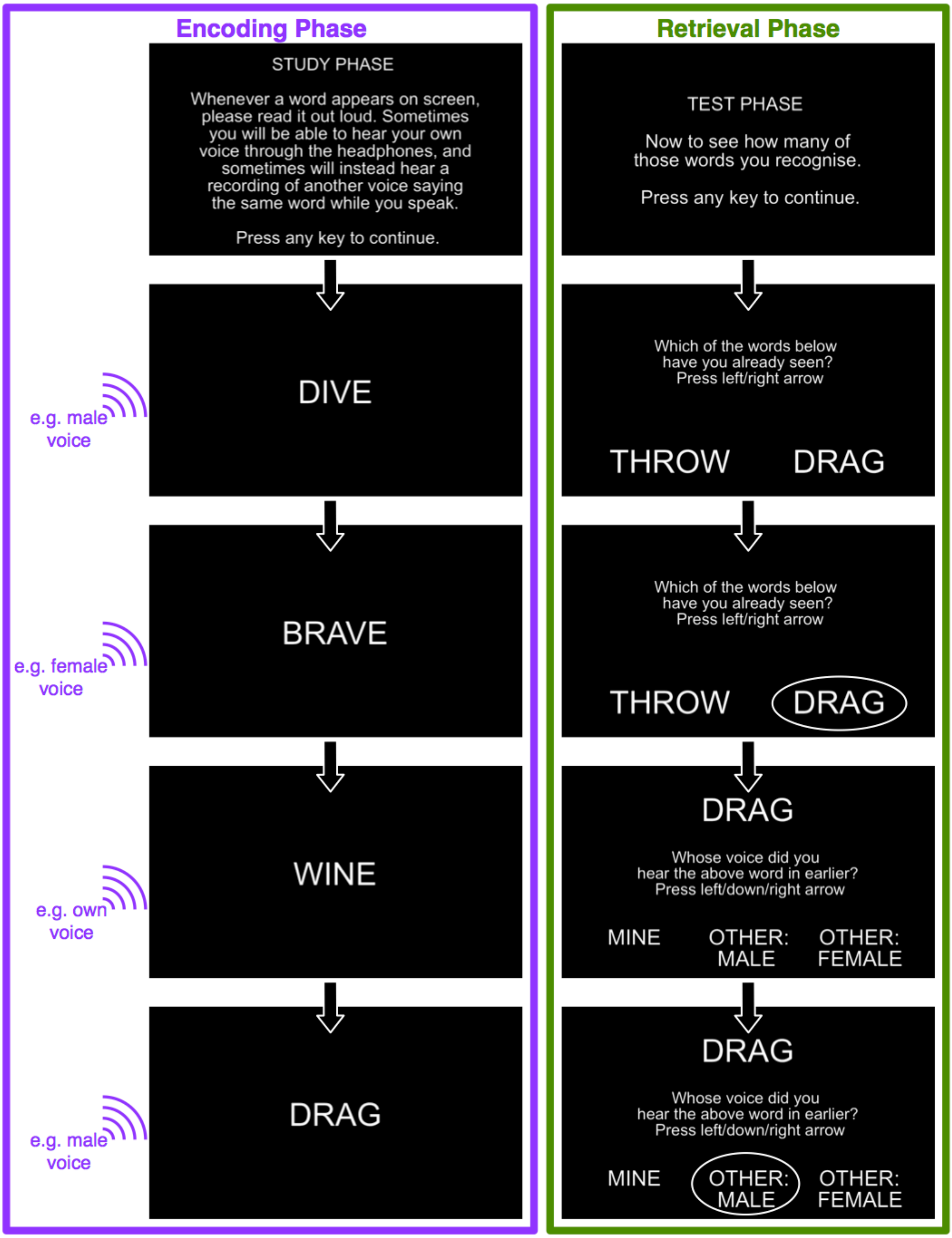

Supplement: Supplementary file 1 [file Image_1.PDF]
